# Assessing the stability and techno-economic implications for wet storage of harvested microalgae to manage seasonal variability

**DOI:** 10.1186/s13068-019-1420-0

**Published:** 2019-04-08

**Authors:** Lynn M. Wendt, Christopher Kinchin, Bradley D. Wahlen, Ryan Davis, Thomas A. Dempster, Henri Gerken

**Affiliations:** 1 Biological and Chemical Processing Department, Idaho National Laboratory, P.O. Box 1625, Idaho Falls, ID 83415 USA; 2 National Renewable Energy Laboratory, Golden, CO 80401 USA; 3 Arizona State University, Mesa, AZ 85212 USA

**Keywords:** Microalgae, Stabilization, Anaerobic storage, Ensiling, Techno-economic analysis

## Abstract

**Background:**

Seasonal variation in microalgae production is a significant challenge to developing cost-competitive algae biofuels. Summer production can be three to five times greater than winter production, which could result in winter biomass shortages and summer surpluses at algae biorefineries. While the high water content (80%, wet basis) of harvested microalgae biomass makes drying an expensive approach to preservation, it is not an issue for ensiling. Ensiling relies on lactic acid fermentation to create anaerobic acidic conditions, which limits further microbial degradation. This study explores the feasibility of preserving microalgae biomass through wet anaerobic storage ensiling over 30 and 180 days of storage, and it presents a techno-economic analysis that considers potential cost implications.

**Results:**

Harvested *Scenedesmus acutus* biomass untreated (anaerobic) or supplemented with 0.5% sulfuric acid underwent robust lactic acid fermentation (lactic acid content of 6–9%, dry basis) lowering the pH to 4.2. Dry matter losses after 30 days ranged from 10.8 to 15.5% depending on the strain and treatment without additional loss over the next 150 days. Long-term storage of microalgae biomass resulted in lactic acid concentrations that remained high (6%, dry basis) with a low pH (4.2–4.6). Detailed biochemical composition revealed that protein and lipid content remained unaffected by storage while carbohydrate content was reduced, with greater dry matter loss associated with greater reduction in carbohydrate content, primarily affecting glucan content. Techno-economic analysis comparing wet storage to drying and dry storage demonstrated the cost savings of this approach. The most realistic dry storage scenario assumes a contact drum dryer and aboveground carbon steel storage vessels, which translates to a minimum fuel selling price (MFSP) of $3.72/gallon gasoline equivalent (GGE), whereas the most realistic wet storage scenario, which includes belowground, covered wet storage pits translates to an MFSP of $3.40/GGE.

**Conclusions:**

Microalgae biomass can be effectively preserved through wet anaerobic storage, limiting dry matter loss to below 10% over 6 months with minimal degradation of carbohydrates and preservation of lipids and proteins. Techno-economic analysis indicates that wet storage can reduce overall biomass and fuel costs compared to drying and dry storage.
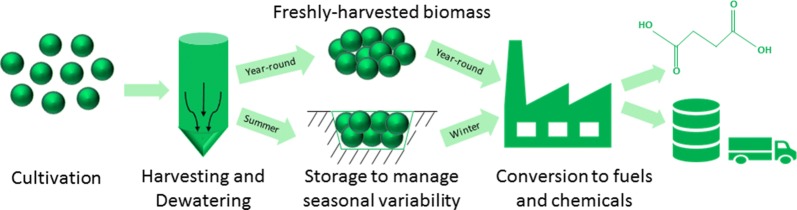

**Electronic supplementary material:**

The online version of this article (10.1186/s13068-019-1420-0) contains supplementary material, which is available to authorized users.

## Background

Microalgae biomass has great potential for use as an agricultural product due to its high conversion rate of sunlight to biomass, its ability to grow in water unsuitable for agriculture (e.g., seawater, saline groundwater), and the potential to utilize carbon dioxide emitted from fossil fuel-based power plants [1]. Furthermore, microalgae biomass has been demonstrated as a feedstock suitable for conversion to bio-based fuels and chemicals [2–4].

Seasonal variation in microalgae biomass production is a well-known challenge when optimizing economics for conversion to biofuels. Harvested biomass levels can vary significantly between summer and winter months, primarily due to the seasonal changes of solar irradiation [5–8]. In algae biofuel conversion designs, seasonal variations result in challenges with maintaining consistent biomass supplies as well as determining the appropriate supply rate on which to base conversion reactor size. One approach to managing seasonal variability is to store a portion of the harvested biomass during the high production months to be used in the winter months such that the biorefinery has a consistent feedstock supply, as described in the conversion designs by Davis et al. and Jones et al. [9, 10]. In these designs, biomass produced in excess of capacity during the summer is preserved, stored, and utilized in the winter months.

Seasonal harvests are a reality for most agricultural products, requiring either immediate use or long-term preservation of the harvested product to maintain quality. One successful preservation approach for any product relies on reducing water activity, or the moisture content at which a product is microbially stable [11]; this is primarily accomplished through drying, although other options include increasing the sugar or salt content in the product. A second approach is to limit the oxygen source in a product such that microbial activity is reduced, and this is commonly accomplished in the food and forage industry through creating conditions favorable for organic acid fermentations. Exclusion of oxygen enables the accumulation of fermentation end products, such as lactic acid, which serves to stabilize the biomass.

Drying has been considered as one approach for stabilization of algae biomass [12–14]. However, preserving algae by drying is energy intensive and can account for over 50% of the total energy demand in algae preprocessing [12]. Wahlen et al. reported that drying harvested algae in a rotary dryer was inefficient, as algae biomass (20% solids) tended to adhere to the walls of the dryer, minimizing surface area for drying resulting in reduced drying rates [15]. Alternative options include solar drying, spray drying, and freeze drying, which are all expensive and incompatible with cost-competitive biofuels [12, 14]. An alternative stabilization method of anaerobic wet storage, i.e., ensiling, is commonly used to preserve forage crops [16], and > 120 million tons of forage-chopped biomass are harvested annually and subsequently stored as silage in the USA [17]. Ensiling relies on initial exclusion of oxygen, followed by bacterial fermentation of soluble carbohydrates to lactic acid. In addition to preservation of forage crops, successful stabilization of macroalgae biomass has been demonstrated through ensiling, and stabilization has been effective for > 200 days [18]. Ensiling has also been proposed for microalgae either stored alone or blended with terrestrial crops to facilitate stable storage [19, 20]. This fermentation can be accomplished by microorganisms present within the algae biomass or through the anaerobic metabolism of the microalgae [21–23]. However, microalgae are still metabolically active post-harvest and thus they present an additional challenge compared to terrestrial biomass or macroalgae. In Wendt et al. artificially created ensiling conditions were used to assess microalgae stability over 1 month of storage at room temperature; *Scenedesmus obliquus* blended with corn stover was stable with total degradation rates under 5%, while *S. obliquus* stored alone incurred total degradation levels of 6–14% [19]. Additional storage studies of microalgae biomass have been limited to a few strains and have been conducted under refrigeration or for short times (less than 1 week) to understand the time frame that the cells can be stored prior to processing the biomass without inducing compositional change [24–26]. However, there are no reported studies regarding how well microalgae can undergo ensiling, and it is unknown how long-term storage at room temperature will impact the major compositional components of the algae including lipids, carbohydrates, and protein.

*Scenedesmus acutus* is an oleaginous microalgae strain that is well suited for high productivity in freshwater sources, and this organism has been explored for its use in algae-based biofuel production [27]. In the present study, the full microbial succession that typically occurs during ensiling was encouraged in *S. acutus*. Multiple anaerobic treatment approaches were investigated to assess the degradation extent and resulting impact on compositional quality of the biomass over 30 and 180 days. Treatments included no additional treatment beyond anaerobic conditions as well as chemical and enzymatic preservative approaches. Techno-economic analysis was performed based on the results of this experiment to investigate the cost-effectiveness of the wet, anaerobic stabilization approach compared to drying using the previously reported frameworks of open-pond raceways and biofuel production utilizing combined carbohydrate fermentation and lipid upgrading [2, 10, 28].

## Results and discussion

### Storage performance

The stability of the harvested *S. acutus* was assessed in wet, anaerobic storage conditions to understand the effect of storage as well as multiple treatments on biomass preservation and resulting quality. Anaerobic conditions with no additives was the simplest treatment to which others were compared. A chemical-based treatment utilizing sulfuric acid was assessed for its potential benefit to preservation through reduced pH and to enhanced lactic acid fermentation. Lower pH limits microbial activity responsible for degradation, and sulfuric acid hydrolyzes carbohydrates to monomeric sugars making them available for fermentation to organic acids and also ensuring the pH remains low. Sulfuric acid treatment has the added benefit of being compatible with algae biomass conversion processes that pre-treat algae biomass with sulfuric acid to obtain fermentable sugars [27]. To enhance stability in an analogous fashion, a commercial enzyme cocktail containing glycosidases, specifically cellulases, β-glucosidases, and hemicellulases, was utilized to release monomeric sugars from complex carbohydrates so that they could be available for fermentation to organic acids, resulting in reduced pH and stable biomass. Glucose oxidase was also evaluated, which couples the oxidation of glucose to the reduction of oxygen, forming hydrogen peroxide and gluconolactone. Glucose oxidase was therefore hypothesized to improve preservation by scavenging oxygen from the storage reactor and by inhibiting microbial growth through the production of hydrogen peroxide.

Storage performance of the treatments after a total of 30 or 180 days is presented in Table 1, with means and lower and upper bounds of 95% confidence intervals presented in Additional file 1: Figure S1. 30-day dry matter loss was lowest for the anaerobic-only treatment at 10.8%, while the losses for alternative treatments were 11.6–15.5%. Total dry matter loss after 180 days was higher in glycosidase and glucose oxidase-treated biomass with 9.7% and 13.2% loss compared to 1.7% and 5% for anaerobic and sulfuric acid-treated samples. Linear regression analysis suggests there is strong evidence that dry matter loss means for all treatments and storage durations were different from zero except anaerobic-only treatment at 180 days. Variation in final dry matter loss levels is seen between samples stored for 30 and 180 days, particularly in the anaerobic and sulfuric acid-treated samples, and this is likely due to slight changes in moisture in extended storage. However, the most notable result of this experiment was that significant additional loss did not occur over this time period for any treatment. These results are important when considering that storage must span a 6-month period between high-productivity summer months and low-productivity winter months.

**Table 1:** Storage performance of *Scenedesmus acutus* biomass after 30 or 180 days of wet anaerobic storage

Treatment^a^	Length of storage (days)	Dry matter loss (%, db)	Material pH	Total organic acids (%, db)^b^
Anaerobic	30	10.8 ± 3.7	4.2 ± 0.06	15.8 ± 4.3
	180	1.7 ± 1.6^c^	4.5 ± 0.06	17.4 ± 4.7
Sulfuric acid (0.5%, db)	30	15.5 ± 1.4	4.2 ± 0.02	17.6 ± 1.8
	180	5.4 ± 4.2^c^	4.6 ± 0.4^c^	16.3 ± 4.7
Glycosidase	30	11.6 ± 2.0	5.0 ± 0.21	10.5 ± 0.7
	180	9.7 ± 1.1	4.4 ± 0.04^c^	19.2 ± 0.6
Glucose oxidase	30	15.1 ± 0.5	5.1 ± 0.1	14.8 ± 1.4
	180	13.2 ± 1.0	4.7 ± 0.2	17.5 ± 3.4

^a^All treatment conditions were stored anaerobically in the dark at 20% solids. Mean (*n* = 3) and standard deviation are presented

^b^Total organic acids refers to the sum of succinic, lactic, formic, acetic, propionic, isobutyric, butyric, isovaleric, and valeric acids present after storage

^c^Represent statistically significant difference between 30 and 180 days treatments based on likelihood probability analysis using 5% confidence intervals

**Fig. 1:**
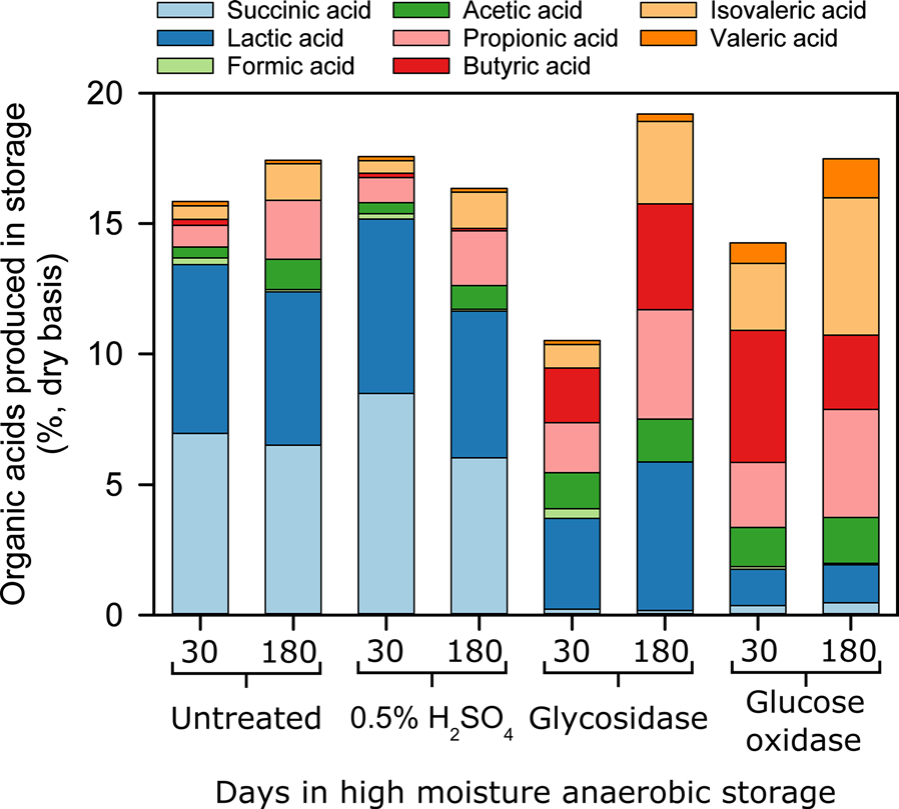
Composition of organic acids produced after storage of microalgae biomass

Organic acid production as a result of storage resulted in 10.5–14.8% dry basis (db) total acids over 30 days depending on treatment, and only in the case of glycosidase treatment did organic acids change significantly over the 180 days storage duration (mean difference is 8.69) (Table 1). However, the composition of organic acids produced in storage was influenced by experimental conditions (Fig. 1). Lactic acid was prevalent in anaerobic and sulfuric acid-treated samples (6.5 and 6.7%, respectively) after 30 days of storage, a trend consistent with well-ensiled herbaceous biomass [16]. Lactic acid was slightly reduced in extended storage in these treatment conditions but was still prevalent at 5.6–5.9%. Alternatively, glycosidase treatment resulted in 3.5 and 5.7% lactic acid after storage at 30 and 180 days, whereas glucose oxidase treatment resulted in 1.4% lactic acid over the storage duration. A substantial amount of succinic acid accumulated in anaerobic and sulfuric acid-treated samples (7.0 and 8.5%, respectively), but not in the enzyme-treated samples (< 0.5%). Succinic acid is a valuable intermediate chemical and is widely recognized as a top value-added product from biomass [29, 30]. Efforts within the biofuels community are underway to develop methodology to convert lignocellulosic and whole algae biomass to succinic acid [2, 27, 31–33], and delivering algae biomass to a refinery with significant levels of succinic acid is a promising option for increasing the value of the biomass.

The storage conditions that incorporated enzyme treatments for preservation resulted in a distinct organic acid profile compared to the anaerobic and sulfuric acid approaches to preservation. The reduction of lactic and succinic acid in the enzyme-treated samples was countered by significant formation of butyric (2.1–5.3%), propionic (1.9–4.2%), and isovaleric acids (0.9–5.3%) during storage. The prevalence of butyric acid in the enzyme treatments could be the result of proliferation of Clostridia species, whose growth may be enabled by the higher pH of 5.0–5.1 for these experimental conditions after 30 days compared to a pH of 4.2 in anaerobic and sulfuric acid-treated samples. Butyric acid formation during ensilage of herbaceous biomass destined for animal feed is discouraged since it results in a feed unpalatable for livestock [34]; however, butyric acid formation in biomass destined for fuel production may have economic advantages if combined with other carboxylic acid fermentations [35]. Propionic and isovaleric acid production in all treatments appeared to be time dependent, experiencing an approximate two- to threefold increase between 30 and 180 days storage depending on treatment. This increase is most prevalent in enzyme-treated samples, likely a result of continued enzyme activity between 30 and 180 days of storage and the corresponding loss of dry matter described above. It is possible that these changes in the enzyme-treated samples are a result of this algae batch being distinct from the algae batch used for anaerobic and acid treatments. Although growths were conducted sequentially in an identical manner, potential changes in bacterial communities could have an unknown effect. Regardless, the excessive loss of dry matter suggests that glycosidase and glucose oxidase treatments have limited applicability for long-term preservation of algae biomass.

The biochemical composition of algae biomass is an important consideration when determining potential fuel yields for biofuel production. Changes in biochemical composition of stored algae were treatment dependent, as shown in Table 2. Lipid content remained constant for the anaerobic and sulfuric acid treatments at 10–10.7%. This is consistent with reported lipid stability in *Tetraselmis suecica* stored at 4 °C over a 90-day period, and *Nannochloropsis salina* stored at 4 °C and 40 °C over a 6-day period [24, 25]. In contrast, the lipid content increased as a fraction of the total biomass for the glycosidase (11.8–12%) and glucose oxidase treatment (13.5–13.7%) after 30 and 180 days of storage due to the loss of other biomass constituents. A detailed analysis of lipid composition (fatty acid) is presented in the Supplemental Information (Additional file 1: Figure S2). Slight differences in lipid composition were observed as a result of glycosidase treatment, specifically in C16 and C18 lipids; however, major differences were not observed as a result of any storage treatment. Protein content for anaerobic 30-day and sulfuric acid 30- and 180-day treatments increased during storage from 32.1% to a high of 34.4% (180 days, sulfuric acid). Differences however, were not substantial and were within 5–7% of the initial biomass. In contrast, enzymatically treated biomass experienced substantial enrichment in protein content and after 180 days protein content increased from an initial value of 32.7% to 39.5% and 41% of the total biomass for glycosidase and glucose oxidase-treated samples. Overall, the impact on lipid and protein changes are an indication that these components are not degraded in anaerobic storage.

**Table 2:** Biochemical composition of *Scenedesmus acutus* biomass after 30 or 180 days of wet anaerobic storage

Experimental condition	Length of storage (days)	Lipids (%, db)	Protein (%, db)	Carbohydrates (%, db)
*S. acutus*—*t*_0_^1^	0	10.1 ± 0.2	32.1 ± 0.14	38.7 ± 0.4
Anaerobic*	30	10.4 ± 0.4	33.7 ± 0.86	33.0 ± 1.2
	180	10.0 ± 0.2	32.2 ± 0.14	33.5 ± 0.9
Sulfuric acid (0.5%, db)*	30	10.7 ± 0.4	33.7 ± 0.05	28.9 ± 0.2
	180	10.7 ± 0.2	34.4 ± 0.05	29.5 ± 0.1
*S. acutus*—*t*_0_^2^	0	10.0 ± 0.3	32.7 ± 0.09	33.7 ± 0.8
Glycosidase*	30	12.0 ± 0.3	35.9 ± 0.29	24.8 ± 0.3
	180	11.8 ± 0.2	39.5 ± 0.48	18.8 ± 0.01
Glucose oxidase*	30	13.7 ± 0.9	38.0 ± 0.05	21.8 ± 0.6
	180	13.5 ± 0.1	41.0 ± 0.09	13.9 ± 0.5

* Biological replicates (*n* = 3) were combined to accommodate all analyses. Variation is represented by standard deviation of analytical replicates (lipids, *n* = 3; protein *n* = 3; carbohydrates *n* = 2)

^1,2^Harvest 1 and 2

The predominant change in biomass composition during wet anaerobic storage occurred in carbohydrates. Carbohydrate content of stored algae biomass was reduced compared to the initial material (Table 2). This is anticipated in ensiling as accessible carbohydrates are fermented to organic acids under the anaerobic conditions in storage, resulting in reduced pH and increased inhibitory organic acids. Similar decreases in carbohydrates in microalgae have been noted during post-illumination respiration of algae and are attributed to dark respiration [36, 37]. Typically, in ensiling of herbaceous biomass, organic acids accumulate until they reach a threshold inhibitory concentration (occurring around pH 4.5), at which point carbohydrate content remains stable. This can be observed in the anaerobic untreated and sulfuric acid treatments where initial carbohydrate content (38.7%) after 30 days in storage was reduced to 33% and 28.9%, respectively. Additional carbohydrates were not consumed during the next 150 days in storage. The fate of carbohydrates after glycosidase and glucose oxidase treatment was much different. Substantial loss from 33.7 to 24.8% and 21.8% was observed after 30 days of storage in glycosidase and glucose oxidase, respectively. Additional degradation of carbohydrates continued during extended storage and resulted in 18.8% and 13.9% carbohydrates remaining as a result of the respective enzymatic treatments.

Changes in carbohydrate composition can be better understood by assessing the fate of each monomer as a result of storage (Table 3). Each treatment condition experienced large changes to both glucose and galactose fractions, with minor changes in mannose content. Reduction of glucose and galactose within anaerobic or sulfuric acid-treated biomass occurred, but did not reduce further with increased storage time. Mannose content was either constant or slightly enriched in these samples, which indicates that it was not consumed during lactic acid fermentation. Unlike anaerobic and sulfuric acid-treated biomass, carbohydrate consumption in enzyme-treated biomass underwent a more severe reduction, a trend which increased with time. Glucose, galactose and mannose were all reduced as a result of glycosidase treatment, likely from the conversion of complex carbohydrates into simple sugars that were then easily utilized as a carbon source. Glucose oxidase-treated algae biomass underwent a reduction in glucose, galactose, and mannose fractions, as these sugars are all substrates for glucose oxidase [38]. In summary, carbohydrate composition was modified as a result of anaerobic storage, with the largest differences experienced in the enzyme-treated conditions.

**Table 3:** Carbohydrate composition of *Scenedesmus acutus* biomass after 30 or 180 days of wet anaerobic storage

Experimental condition	Length of storage (days)	Glucan (%, db)	Galactan (%, db)	Mannan (%, db)
*S. acutus*—*t*_0_^1^	0	26.9 ± 0.04^a^	3.7 ± 0.3^a^	8.1 ± 0.1^a^
Anaerobic*	30	22.8 ± 1.1^b^	1.9 ± 0.2^b^	8.3 ± 0.1^ab^
	180	23.5 ± 0.7^b^	1.3 ± 0.05^b^	8.7 ± 0.2^b^
Sulfuric acid (0.5%, db)*	30	19.2 ± 0.01^c^	1.3 ± 0.1^b^	8.4 ± 0.05^ab^
	180	19.9 ± 0.04^c^	1.3 ± 0.04^b^	8.2 ± 0.01^a^
*p* value		< 0.001	< 0.001	0.018
*S. acutus*—*t*_0_^2^	0	21.9 ± 0.4^a^	3.1 ± 0.1^a^	8.7 ± 0.3^a^
Glycosidase*	30	15.2 ± 0.3^b^	0.9 ± 0.01^b^	8.7 ± 0.01^a^
	180	10.7 ± 0.01^c^	1.0 ± 0.00^b^	7.1 ± 0.02^b^
Glucose oxidase*	30	12.5 ± 0.2^d^	1.3 ± 0.1^b^	8.1 ± 0.2^a^
	180	5.7 ± 0.2^e^	1.2 ± 0.0^b^	7.0 ± 0.3^b^
*p* value		< 0.001	< 0.001	< 0.001

* Biological replicates (*n* = 3) were combined to accommodate all analyses. Variation is represented by standard deviation or analytical replicates (*n* = 2). Letters represent statistically distinct values as determined by Tukey’s test

^1,2^Harvest 1 and 2

Changes to algae biomass occurring in storage were also assessed by bomb calorimetry, proximate and ultimate analysis (Additional file 1: Table S3). Notably, all samples after 180 days of storage had increased amount of carbon and decreased amount of oxygen as a fraction of the total biomass. This change in elemental composition is further reflected in an overall increase in the energy density of stored biomass; the higher heating value (HHV) for all treatments increased after 180 days of storage relative to the initial value. Similar changes in elemental composition and energy density have been demonstrated in algae stored anaerobically [19]. The increase in energy density should translate into higher fuel yields in thermochemical conversion on a per unit of biomass basis, although total yield would be expected to be less as a result of material losses in storage. Additional experiments would be required to fully understand the impact of storage on thermochemical conversion.

### Techno-economic analysis

**Fig. 2:**
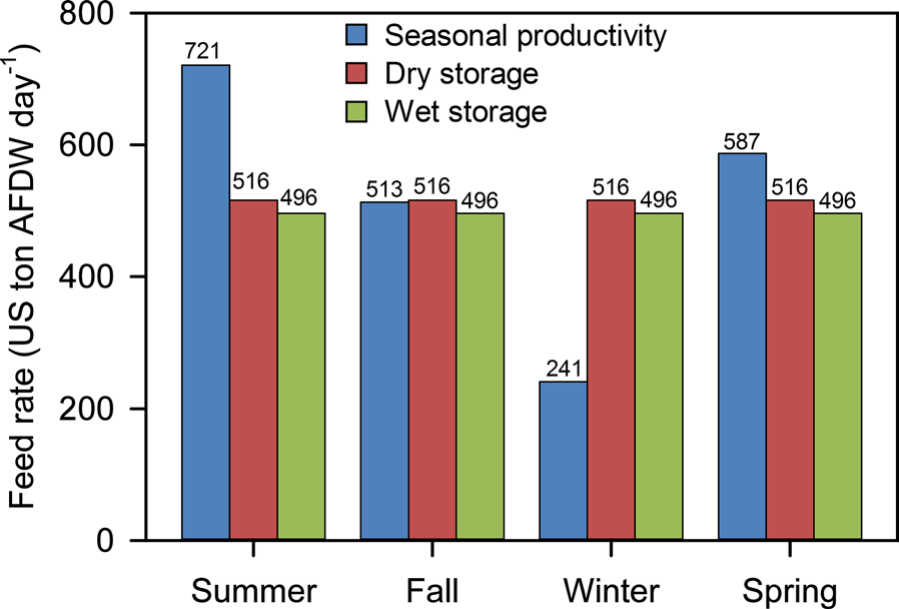
Seasonal biomass productivity and corresponding conversion feed rate for dry and wet storage scenarios

**Fig. 3:**
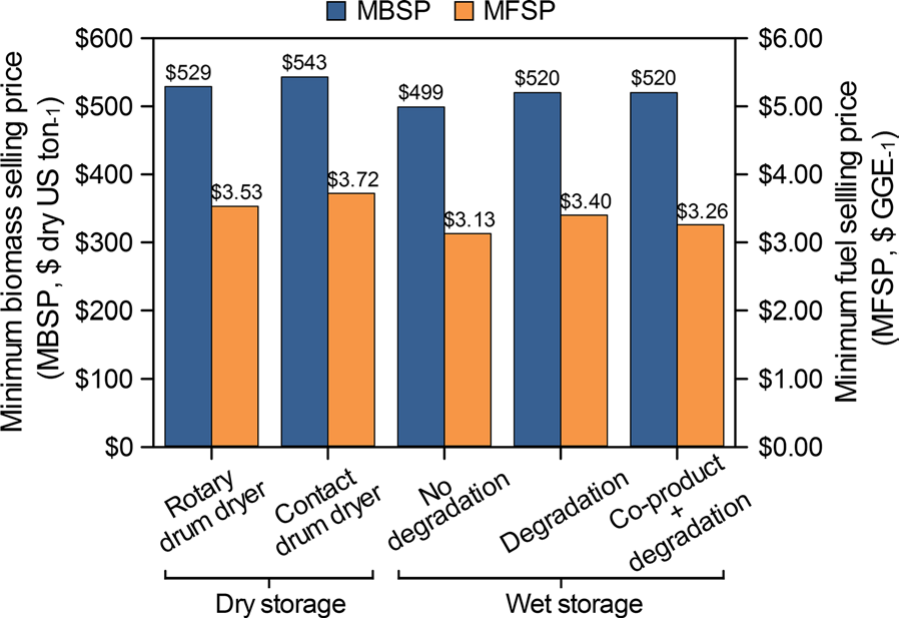
MBSP and MFSP results of TEA models for all dry and wet storage scenarios evaluated

Techno-economic analysis (TEA) was performed to evaluate the feasibility of a series of conversion scenarios where a fraction of microalgae is sent to storage (dry or wet) in high-productivity warm months and drawn from storage in low-productivity cold months to supply the conversion facility with a consistent feed rate of microalgae year-round, thus reducing or eliminating underutilized equipment capacity. Figure 2 illustrates the difference in seasonal productivity at the algae farm relative to consistent capacity sent through the conversion facility for both dry and wet storage scenarios. The values for dry and wet storage represent the amount of algae sent to the conversion facility to maintain consistent capacity at the conversion facility throughout the year. The design cases for algae biomass cultivation [28] and combined fermentation and lipid upgrading to fuels and chemicals [10] developed at National Renewable Energy Laboratory (NREL) were used to calculate minimum biomass selling price (MBSP) and minimum fuel selling price (MFSP), respectively, for each of the dry and wet storage scenarios. In the scenarios presented here, the fuel product is mostly renewable diesel blendstock, with some naphtha, while the chemical coproduct is succinic acid. Table 4 and Fig. 3 summarize the results of the TEA evaluations.

**Table 4:** TEA results for all dry/wet storage scenarios evaluated

Design scenario	Biomass yield, MM US ton/year	MBSP, $/ton	Fuel yield, MM GGE/year	Fuel yield, GGE/dry US ton algae	Succinic acid yield, ton/year	Succinic acid yield, lb/dry US ton algae	MFSP, $/GGE
Dry storage, rotary drum dryer	0.188	$529	13.8	73.7	66,509	708	$3.53
Dry storage, contact drum dryer	0.188	$542	13.8	73.7	66,509	708	$3.72
Wet storage, no degradation	0.188	$499	13.8	73.7	66,509	708	$3.13
Wet storage with degradation	0.181	$520	13.4	74.3	63,660	703	$3.40
Wet storage with degradation and succinic acid credit	0.181	$520	13.4	74.3	65,048	719	$3.26

*GGE* gasoline gallon equivalent, *MM* million

The dry storage, rotary drum dryer design scenario reflects the previously established design basis as utilized in recent TEA models [10, 28], which place drying and storage operations as the first step in the conversion model. To quantify the impact of storage degradation losses on the resulting MBSP, drying and storage operations were moved to the algae farm model. As a result, the original $491/ton basis reported in previous NREL algae biomass design reports [28] increased to $529/ton. Given that this is largely an artifact of where drying and storage costs are allocated, the $529/ton value reported here is nearly equivalent to the $491/ton value reported in the prior work with respect to downstream implications on fuel costs (although in the present work, storage tanks for the dried biomass are explicitly included in the cost which further increases direct capital expenses, relative to the prior work which implicitly assumed the storage tanks to be included in the indirect facility cost factors). MBSP for the contact drum dryer dry storage design scenario increased as a result of using contact drum dryers instead of rotary drum dryers, but may be viewed as a more realistic design if the use of algae biomass dryers were employed. Rotary drum dryers work by bringing material to be dried into direct contact with a heated gas while rotating inside a cylinder. The rotating cylinder enhances drying by lifting particles with fins on the inside of the rotating drum and then showering the material through the hot gas stream as it falls back to the bottom of the cylinder. Recent work indicates rotary drum dryers may not effectively dry algae biomass, as sludge-like materials similar to dewatered algae have a tendency to adhere to surfaces in the dryer and agglomerate, resisting the lifting and falling action that increases drying surface area. In addition, drying occurs more rapidly at the material surface relative to its interior, forming an outer crust that further interferes with removing moisture from the interior [15]. Based on this, contact drum dryers were considered as a potentially more suitable candidate, as they are designed to handle material similar to algae paste, although the suitability for this choice would still require experimental verification and is currently not well understood. Contact drum dryers conduct heat to material applied on the surface of a heated rotating drum. The biomass adheres to the outside of the drums and is scraped off as the drums continue to rotate. Replacing rotary drum dryers with contact drum dryers in the dry scenario translated to an increase in modeled MBSP from $529 to $542/ton.

Next, TEA was used to assess wet storage, evaluating one scenario with no storage degradation and two scenarios with degradation based on the experimental results from the anaerobic, no additive cases, both including and excluding a coproduct credit for succinic acid produced in storage on the final fuel costs. Modeled MBSPs are lower for all three wet storage scenarios compared to the dry storage scenarios. MBSP in the wet storage with no degradation loss scenario is the least costly of all scenarios because it does not incur drying costs or degradation losses. In the remaining two design scenarios, storage degradation losses are included, which increased MBSP as production and storage costs remained unchanged but were allocated over a lower biomass yield. Notably, modeled MBSPs are lower for all wet storage scenarios compared to the dry scenarios even after including degradation losses. This is reflective of relatively high capital costs for both the dryers and aboveground storage as well as operating costs for natural gas consumed in the dryers, all of which are replaced by more simplistic in-ground covered pits for wet storage.

As can be seen in Table 4, biomass yields from the microalgae farm are identical for all scenarios when storage degradation losses are not considered. Fuel yield per year and per dry US ton of algae directly reflect whether storage losses were included in the analysis. An interesting finding reported in Table 4 is the fuel yield per dry ton of algae for the last two design scenarios, both wet storage with degradation losses. The fuel yield per dry ton of algae increases from 73.7 to 74.3 GGE/ton biomass relative to the other storage scenarios due to storage degradation losses disproportionately occurring in the carbohydrate fraction of the biomass rather than the lipid/free fatty acid fraction (Table 5). As a result, the fuel yield per ton of algae increases slightly (although the dry ton algae yield is reduced). Again, expecting no changes due to degradation losses is not likely realistic for wet microalgae storage and is not consistent with experimental results reported here, but it offers value by allowing for isolation of cost impacts due only to capital and operating costs for wet and dry storage. Assuming no degradation losses also provides a best-case scenario for strategies aimed at mitigating storage degradation losses, such as selecting different algae strains or applying stabilizing agents.

**Table 5:** Input compositions to TEA models based on baseline (raw) and wet storage composition

	Raw algae	Wet storage algae
Solids content (wt%)	20	20
Algae composition (wt%)		
Protein	13.2	14.2
Free fatty acids	26.0	27.5
Ash	2.4	3.1
Fermentable carbohydrates	47.8	46.2
Non-fermentable carbohydrates	3.2	1.7
Glycerol	3.0	3.0
Non-fuel polar lipid impurities	2.8	2.8
Cell mass	1.6	1.6
Sum	100.0	100.0
Storage losses^a^		22.8%
Acid produced per kg of whole algae (after storage)		
Succinic acid, kg		0.090
Lactic acid, kg		0.083

^a^Based on total dry matter loss, succinic acid, and lactic acid formation in anaerobic-only treatment after 30 days of storage

The succinic acid yield for the first three design scenarios is 66,509 tons/year, and 708 lb per dry ton of algae (Table 4). The storage degradation losses included in last two design scenarios cause succinic acid yield to decrease to 63,660 and 65,048 tons/year, respectively, particularly because carbohydrates are most preferentially degraded. The only difference between the last two design scenarios is the addition of succinic acid produced as a storage degradation product to the succinic acid intentionally produced in the conversion process. This translates to a $0.14/GGE decrease in MFSP between the last two scenarios, primarily driven by the high coproduct value of succinic acid, $2.14/kg ($0.97/lb), which illustrates the significant reduction in MFSP that can be realized by capitalizing on high-value coproduct opportunities. This result also suggests that a small amount of degradation during wet storage may translate to a net MFSP benefit, provided the degradation products are valuable and can be recovered. This approach has been documented with fermentation products beyond succinic acid including lactic, acetic, and propionic acids [39].

MFSP was calculated based on the MBSP, fuel and succinic acid yields, and capital and operating costs (Table 4). Due to the fact that rotary drum dryers may not effectively dry algae biomass, the most realistic dry storage scenario is likely the dry storage with contact drum dryers. The MFSP for this scenario is $3.72/GGE, higher than the $3.53/GGE result for the dry storage with rotary drum dryer scenario. The most realistic wet storage scenario (as reflective of the currently available data) is the wet storage with degradation losses scenario: storage in covered, belowground ponds, including storage losses and composition changes, while not giving credit to final MFSPs for succinic acid production during wet storage because succinic acid production is not guaranteed. The MFSP of $3.40/GGE for this scenario is slightly less than the dry storage with rotary drum dryer scenario ($3.53/GGE), but considerably less than the most realistic dry storage scenario (contact drum dry storage scenario at $3.72/GGE). Thus, overall this preliminary analysis indicates that wet storage appears to compare favorably to dry storage when all elements are evaluated consistently. The last design scenario (wet storage in covered, belowground ponds, including storage losses and composition changes, while giving credit to final MFSPs for succinic acid production) can be considered a more optimistic scenario if the downstream conversion process is configured to target coproduction of the same types of organic acids being evolved during storage.

The difference between the third and fourth design scenarios (wet storage with and without degradation losses), with MFSP estimates of $3.13 and $3.40/GGE, respectively, demonstrate that storage losses do not affect final MFSP as significantly as may be expected considering 22.8% of algae biomass is lost to degradation products during wet storage. This can be explained in that only 16.2% of annual algae biomass produced is diverted to wet storage, therefore only 3.7% of annual algae biomass produced over the year is lost during wet storage degradation. It should be noted that these values (percent diverted and percent lost) are based on a targeted seasonal productivity variability of 3:1 (i.e., maximum versus minimum seasonal cultivation productivity). Higher variability will require more algae being diverted to storage annually, thus increasing the percent of algae lost to degradation. Another factor that may contribute to the fractional degradation losses may be the storage duration. This analysis is based on the 30-day anaerobic-only wet storage experiments; however, data from 180-day storage experiments indicate degradation losses beyond 30 days are minimal. Additional reduction of storage loss may be possible, as the current results represent one of the first attempts of long-term storage for wet algae and can be likely improved upon.

The primary advantage of utilizing storage to manage seasonal variation in biomass productivity results in the reduction of capital equipment costs as well as the elimination of underutilized equipment. Davis et al. previously demonstrated a reduction in MFSP by 4–6%, depending on the biomass composition, when dry storage was utilized relative to designing all equipment to accommodate peak seasonal flows directly from biomass production [10]. Both wet and dry storage can eliminate underutilized equipment in the conversion facility, but drying and conveying equipment must be sized to accommodate the maximum productivities during summer months. It is unavoidable that all drying capacity will sit idle for about half the year, while much of the drying capacity will be underutilized the rest of the year. Therefore, dry storage does not eliminate all underutilized equipment capacity. The dry storage scenario still may lack two additional factors that could further increase costs, namely the potential for at least some storage degradation losses (not currently accounted for) and also potential requirements for climate control given that storage is envisioned to occur during summer months in the US Gulf Coast region with high humidity (this is also not accounted for, and could potentially incur significant cost and life cycle assessment penalties if it were required). Biomass storage losses also require that a larger amount of algae be stored seasonally to achieve fixed biomass throughputs downstream through the conversion facility, and this is only reflected in the wet storage scenarios presented in this TEA.

The TEA findings in this study reiterate the important observation that seasonal variability in algae cultivation productivity leads to either algae degradation losses for wet storage, or underutilized drying capacity for dry storage, both of which increase MFSP. Additionally, greater seasonal variability in algae productivity leads to greater storage requirements and costs, further increasing MFSP. Therefore, the results of this analysis further validate the conclusions of previous analyses [10] that emphasize the importance of improving winter productivity to the extent possible, either through strain rotation or other strategies (although recognizing it is not likely that winter productivity can ever be improved to match summer productivity, at least for outdoor cultivation systems in the USA, given the lower solar irradiation available in the winter months). Finally, it should also be noted that wet algae storage research is in the nascent stages of development, and improvements are possible that may reduce losses and minimize composition changes.

## Conclusion

This paper describes approaches for stabilization of freshly harvested microalgae to manage seasonal variation due to algae biomass productivity changes. Storage performance in *S. acutus* was assessed after 1 and 6 months. Dry matter losses over 6 months ranged from a low of 1.7% (anaerobic treatment) to a high 13.2% (glucose oxidase treated). Changes in biochemical composition were characterized by reduction of carbohydrates and preservation of lipids and protein. Anaerobic-only and sulfuric acid treatments resulted in the lowest dry matter loss and fewest compositional changes. Techno-economic analysis was performed to compare wet storage to drying and dry storage costs on overall fuel selling price based on a conversion approach that converts carbohydrates to chemicals and upgrades lipids to fuels. Drying and aboveground storage vessels considerably increase MFSP due to capital costs as well as natural gas required for drying. Mass losses and composition changes due to algae degradation during wet storage have relatively less impact on MFSP than expected, though not insignificant. A wet storage approach that considered storage degradation was shown to reduce MFSP to $3.40/GGE compared to $3.72/GGE for drying using a contact drum dryer. Succinic acid produced during wet storage may also be captured downstream to further reduce MFSP to $3.26/GGE, suggesting that coproduct formation in storage could not only stabilize biomass, but also reduce final fuel production costs. Overall, wet storage offers significant potential as a cost-effective approach for managing seasonal variability and maintaining a stable feedstock source for conversion. Future research in this area is warranted to understand the impact of these storage approaches on additional microalgae species as well as to demonstrate the conversion efficacy of the storage approach utilized in the TEA presented in this study.

## Methods

### Algae cultivation

Algae cultivation was performed at the Arizona Center for Algae Technology and Innovation in Mesa, AZ, in a containment greenhouse. Two batch cultures were grown sequentially in December and January. *Scenedesmus acutus* LRB0401 was inoculated at 0.05 g/L and grown in BG-11 medium. Algae was cultured in 110 L vertical flat panel photobioreactors with a 2-in. light path using natural lighting (natural diurnal light dark periods). High temperatures averaged 20 °C and low temperatures averaged 7 °C during both batch runs. Each batch culture was grown over a 3-week period and harvested when culture density reached 3 g/L. The algae biomass was dewatered at 1800×*g* through Lavin 20-1160V Centrifuges (AML Industries, Inc, Warren, OH) with a flow rate of approximately 2 L/min. Dewatered algae were placed into Ziploc^®^ bags, stored in a cooler on ice, and shipped overnight to Idaho National Laboratory.

### Storage experiments

Algae biomass storage experiments were initiated by mixing algae with appropriate materials to achieve the desired experimental conditions. Triplicate storage reactors were used for all treatments. The simplest of the experiments (anaerobic treatment) involved adding algae biomass (20% solids) to 2 oz. jars fitted with an airtight lid and a through-lid bulkhead adapter (SS-400-R1-4, Swagelok, Solon, OH), which accommodated the connection of a gas collection bag (P/N 262-01, SKC, Eighty Four, PA) with silicon tubing. For sulfuric acid treatment, algae biomass was first mixed with a sufficient amount of 72% sulfuric acid to achieve a loading of 0.5% acid (wt/wt, db). For both the anaerobic and acid treatment, algae biomass was added up to the top of the jar (2 oz) to minimize headspace and facilitate the establishment of an anaerobic atmosphere.

The second algae biomass harvest was utilized for enzyme treatments. Algae biomass was mixed with either glycosidase (Cellic Ctec2, Novozymes, Franklinton, NC) at 400 µg/g (db) or glucose oxidase (P/N G7141, Millipore Sigma, St. Louis, MO) at 11 U/g (db) and then added to 4 oz. jars with airtight lids fitted with a through-lid bulkhead tube adapter and a ball valve (P/N B-43S4, Swagelok, Solon, OH). These jars were only filled partway to permit material expansion. After filling, jars were repeatedly evacuated and filled with nitrogen gas to establish an anaerobic atmosphere. Gas collection bags were then fitted to the ball valve with silicone tubing. Dry matter loss after 30 or 180 days was determined, as described previously [19].

### Analysis of fermentation products

Organic acids present in both fresh and stored samples were extracted using a 1:10 ratio of wet biomass (1 g) to 18 MΩ nanopure water and analyzed in duplicate using high-performance liquid chromatography (HPLC), as described previously [19].

### Lipid analysis

Algae biomass was lyophilized and ground by mortar and pestle. Lipid content and fatty acid composition of unstored and stored algae biomass were determined as described by Van Wychen et al. [40]. Each sample was analyzed in triplicate. Each experimental replicate was analyzed for anaerobic and acid-treated biomass, and a composite of triplicate storage experiments was formed using lyophilized glycosidase and glucose oxidase-treated algae biomass.

### Carbohydrate composition

Total carbohydrate content of unstored and stored (composite of triplicate biological replicates) algae biomass was determined by the two-step process described in Van Wychen et al. [41], with all samples measured in duplicate. A composite of triplicate storage experiments was formed using lyophilized algae biomass, and an exception to this occurred in the anaerobic, 30-day storage sample, where all triplicate storage experiments were analyzed. Carbohydrates were first hydrolyzed with 72% sulfuric acid, diluted, and autoclaved at 121 °C for 60 min. Duplicate samples were quantified for monomeric sugars by high-performance liquid chromatography (Agilent, Santa Clara, CA) using a Bio-Rad guard column (P/N 125-0118, Bio-Rad Laboratories, Hercules, CA), Shodex Sugar SP0810 column (P/N F6378105, Showda Denko America, Inc., New York, NY) and refractive index detector.

### Proximate and ultimate analysis

Unstored and stored (composite of triplicate biological replicates) lyophilized samples were run in triplicate for proximate, elemental CHN, and elemental S analyses as described previously [19]. Proximate analysis was preformed using a LECO TGA701 Thermogravimetric Analyzer (St. Joseph, MI, USA) following ASTM D 5142-09 [42] to determine moisture, volatile, ash, and fixed carbon content. Ultimate analysis was performed using a LECO TruSpec CHN and S add-on module following ASTM D5373-10 [43, 44] and ASTM D4239-10 [42], respectively, to determine elemental carbon, hydrogen, nitrogen, and sulfur concentrations. Protein content was determined by multiplying the elemental nitrogen content by a conversion factor of 4.78 [45].

### Statistical analysis

Averages and one standard deviation were calculated for triplicate biological replicates in storage experiments. Linear regression was used to model changes in dry matter loss, pH, and organic acids produced in storage as a function of all treatment and time point combinations. Approximate 95% confidence intervals were found using profile likelihoods assuming constant variation among groups.

### TEA approach and assumptions

The techno-economic analysis (TEA) consists of modeling the material and energy balances in Aspen Plus (Aspen Technology, Inc., Bedford, Massachusetts), followed by a discounted cash flow rate of return analysis (DCFROR) in Microsoft Excel to determine minimum fuel selling price given a net present value of zero for a 10% internal rate of return. The Aspen Plus model is divided into two sections: an algae cultivation model (algae farm) that produces algae biomass at 20 wt% solids after dewatering, followed by a conversion model that converts algae biomass to diesel blendstock and coproducts. The general concepts for both processes is explained in detail in Davis et al. [10, 28]; however, a brief explanation of the combined process is provided here.

A model strain of *Scenedesmus acutus* is grown in unlined open ponds, occupying a total pond and cultivation area of 5000 wetted acres (2023 ha) excluding the inoculum system. The inoculum system consists of three sequential stages: a closed photobioreactor (PBR) system is used for the first stage of inoculum growout, followed by covered ponds of significantly larger cultivation footprint than the PBR’s, and then lined ponds with a larger footprint than the covered pond stage. The main production ponds are 10-acre in-ground, uncovered ponds. However, capital and operating cost for the ponds are based on the average of estimates from four slightly different raceway and serpentine pond designs. Purified CO_2_ from flue gas carbon capture (i.e., amine scrubbing, membrane purification, etc. which may be expected to provide CO_2_ at > 99% purity) is sparged into the production ponds during daylight hours. Algae grown in the ponds is harvested at a fixed concentration of 0.05 wt% solids (0.5 g/L) and dewatered to 20 wt% solids (200 g/L, AFDW) in a three-step process consisting of gravity settling, membranes, and centrifugation. Dilute water from the dewatering process is recirculated to the production ponds. The dewatered algae stream is then sent to a co-located conversion facility for conversion to fuels and coproducts. Depending on the time of year and seasonal productivity, a portion of the harvested algae is either diverted to storage, or algae are drawn from storage, as shown in Fig. 2. In the baseline cases of previously published design reports by Davis et al. [10, 28], the algae diverted for storage are first dried. An alternative storage method, wet storage, is the focus of this analysis.

The conversion process from algae to fuels and coproducts is based on a modified version of NREL’s published “Combined Algae Processing” (CAP) pathway [10], wherein the algae biomass is combined with steam and treated with dilute sulfuric acid catalyst at high temperature to hydrolyze the glucan carbohydrates to monomeric sugars and make the biomass amenable for downstream lipid extraction. In the cited base CAP model, the liberated sugars are fermented to ethanol, algae lipids are extracted and converted (hydrotreated) to a renewable diesel blendstock (RDB), and protein is directed to an anaerobic digestion process, where the methane-rich biogas product is combusted in a gas turbine to generate electricity. Flue gas heat is used generate steam for utility demands. Subsequent NREL modeling efforts indicate targeting alternate coproducts from sugar, such as succinic acid rather than ethanol, may produce more favorable overall economic results as a means to ultimately reduce algae fuel costs to economically viable targets in the future [2]. Therefore, in this analysis, sugars are converted to succinic acid, while lipids are again converted to RDB, and protein is again sent to anaerobic digestion. Lipids are isolated using a countercurrent liquid–liquid extraction process with hexane solvent. The solvent–lipid phase is sent to a distillation stripping column to recover the solvent, while the oil is sent to a series of purification steps including degumming, demetallization, and bleaching to remove phospholipids, metals, salts, and other impurities. The purified oil is then sent to hydroprocessing for conversion to diesel-range paraffinic product suitable as a diesel blendstock (RDB) with a small naphtha coproduct. The residual raffinate stream from the lipid extraction step is combined with the oil purification waste stream and sent to anaerobic digestion, primarily used as a means to reclaim carbon via biogas production as well as enabling nutrient recycle to the algae production ponds. The sugar-to-succinic acid process and associated design/economic details are consistent with previously documented TEA research [31].

In the economic analysis, capital and operating costs are estimated for the entire process and a DCFROR is performed in Microsoft Excel to calculate MFSP. The MFSP can be described as the price at which the fuel must be sold to achieve a 10% internal rate of return for the project. The economic analysis was performed in 2014 US dollars. The purpose of this analysis is to compare wet and dry storage; therefore, a thorough explanation of the economic assumptions of the entire process is not warranted here, although the details can be found in Additional file 1: Table S3 and elsewhere [10, 28]. In addition to calculating an MFSP, MBSP is also calculated, which includes the capital and operating costs required to grow and store the algae prior to routing through downstream conversion to fuels.

Experimental data for biomass degradation from the 30-day storage case for the anaerobic-only storage approach was used in this analysis. The mass closure of the data varied from 79 to 92%, although some of the unaccounted mass may be present as minor components that were not measured, such as glycerol, chlorophyll, polar lipid impurities (i.e., sterols and non-fatty acid methyl ester (FAME) lipids), and nucleic acids. These additional components have previously been measured for similar biomass species and compositional profiles and were added accordingly here (unpublished data). Table 5 shows the biomass composition assumed in the TEA model both before and after wet storage. The raw algae composition prior to storage was set consistently with previous TEA modeling work with additional granularity shown in extrapolating lipids characterized as FAME to free fatty acids plus glycerol [28, 46, 47]. From there, the major components (protein, total carbohydrates, ash, and lipids as free fatty acids) were adjusted in the “after storage” case based on the experimental storage data, generally reflecting a degradation in total carbohydrates offset by an increase in remaining protein, lipids, and ash. The minor components (glycerol, lipid impurities, and cell mass) were left unchanged as those components were not tracked in this experimental work and do not contribute appreciably to overall fuel selling price in the model.

While the compositions for whole algae biomass (i.e., when excluding extracellular degradation products) do not appear to change significantly, the wet storage algae composition in Table 5 is distributed over less mass, as 22.8% of total algae mass is lost to CO_2_ and degradation products during wet storage. As the degradation products are not part of the intact algae cell, the whole algae composition in Table 5 is closed to 100% without including those components. Succinic and lactic acid are then added as separate extracellular components based on the mass ratio of acid produced per kg of whole algae remaining after storage (CO_2_ is not shown in this table, but generally is expected to constitute the remaining mass discrepancy from the original biomass before storage degradation). An additional note regarding Table 5 is that the ash content in the wet storage algae composition was set manually to maintain an equal mass of ash in the raw and stored algae.

### Design scenarios and economic assumptions

Five design scenarios were evaluated in total: two using dry storage and three using wet storage. The first dry storage scenario reflects the current design scenario baseline as typically assumed in recent TEA models: dry storage with a rotary drum dryer. The second dry storage scenario assumes a different type of dryer, a contact drum dryer. All dry storage scenarios assume no algae losses or composition changes during storage. The first wet storage scenario assumes no algae losses or composition change during wet storage, primarily to isolate the capital and operating cost impact of wet storage relative to dry storage (and as a “best-case” scenario for controlling degradation losses in the future). The remaining two wet storage scenarios include storage degradation losses and composition changes, with and without credit for succinic acid produced during wet storage. The five scenarios are described in more detail as follows.

In the first design scenario, algae biomass is dried in a rotary drum dryer and conveyed to a covered storage area. Capital costs for conveyors are estimated using Aspen Capital Cost Estimator (ACCE) software [48]. The conveyor is covered, 300 feet in length, and 5 feet wide. Three conveyors are deemed necessary: one to transport dry algae from the dryers to the storage area, a second conveyor to feed the storage vessel, and a third conveyor to move algae from the storage vessel to the conversion facility. Power requirements for each conveyor are estimated at 50 hp, based on previous NREL design reports [49]. The capital costs for large aboveground storage structures, constructed of carbon steel, are estimated using ACCE software and reported in Additional file 1: Table S2; these costs were implicitly assumed to be included in other indirect cost factors under the previously cited design report [10], but are now explicitly accounted for in this work. Two operators were deemed necessary for conveying and storage operations. Additional operating costs for natural gas consumption are reported in Additional file 1: Table S2. This first design is consistent with previous TEA assumptions for this drying operation, with the exception of aboveground storage costs now explicitly included in the direct capital expenses as noted above [10].

The second dry storage scenario is consistent with the first scenario but uses a different type of dryer, a contact drum dryer. The capital costs of contact drum dryers were estimated based on engineering design correlations [50]. Operating costs for the contact drum dryer, mainly natural gas consumption and labor costs, are assumed to be identical to that of the rotary drum dryer.

The third design scenario assumes wet storage in belowground storage pits, but does not consider potential degradation losses or composition changes due to degradation. Capital costs for wet storage containment were based on belowground storage pits, estimated using settling ponds from the Davis et al.’s design report [28] as a proxy for storage pits. Covers were assumed for the storage pits, with capital cost for the covers based on capital cost for cultivation pond liners from the same report. The resulting costs were consistent with a second estimate furnished by an engineering subcontractor for such in-ground pits. The total required storage pit area would cover approximately 5 acres. Alternatively, aboveground steel tank storage vessels were also considered, but the costs for such large storage volumes with wet material would be prohibitive, and we believe the less expensive belowground “covered pit” option is viable for this approach. Transport (i.e., conveyor) costs from the dewatering area to the wet storage area are not estimated, as the algae can still be pumped at 20 wt% solids, and the wet storage area will be located near both the dewatering operations and the conversion facility. An additional operator is added to attend to the wet storage area, relative to the labor costs detailed in the Davis et al. design report [28].

The fourth design scenario is similar to the third scenario but includes storage degradation losses and composition changes due to degradation as documented in Table 5. Succinic acid produced during wet storage is not given credit for final MFSPs in this scenario, as this component may not always be produced depending on algae strain selection and storage conditions, as well as in potentially more optimized approaches wherein lactic acid-producing organisms are inoculated in the storage media as utilized for biomass ensiling (which then may limit succinic acid evolution).

The fifth design scenario is similar to the fourth scenario, but succinic acid produced during wet storage is added to the succinic acid yields produced downstream through sugar fermentation, thus giving credit for succinic acid produced during wet storage in resulting final coproduct outputs and associated fuel selling price calculations.

## Additional file

**Additional file 1.** Additional information on composition of experiments and capital and operating costs associated with dryers.

## Abbreviations

MFSP: minimum fuel selling price
GGE: gallon gasoline equivalent
db: dry basis
TEA: techno-economic analysis
MBSP: minimum biomass selling price
RDB: renewable diesel blend
CAP: combined algae processing
DCFROR: discounted cash flow rate of return
