# Supplementary material for: Assessing the stability and techno-economic implications for wet storage of harvested microalgae to manage seasonal variability
Source: Biotechnol Biofuels. 2019 Apr 8;12:80. doi: 10.1186/s13068-019-1420-0 (PMC6452513; doi:10.1186/s13068-019-1420-0)
Supplement: Supplementary file 1 — Additional file 1. Additional information on composition of experiments and capital and operating costs associated with dryers. [file 13068_2019_1420_MOESM1_ESM.docx]

Assessing the Stability and Techno-Economic Implications for Wet Storage of Harvested Microalgae to Manage Seasonal Variability

Lynn M. Wendt^1†*^, Christopher Kinchin^2†^, Bradley D. Wahlen^1^, Ryan Davis^2^, Thomas Dempster^3^, Henri Gerken^3^

^1^ Idaho National Laboratory, Biological and Chemical Processing Department, Idaho Falls, ID 83415

^2^ National Renewable Energy Laboratory, Golden, CO 80401

^3^ Arizona State University, Mesa, AZ 85212

†These authors contributed equally to the manuscript.

*Corresponding author:

Lynn.Wendt@inl.gov

Idaho National Laboratory

P.O. Box 1625

Idaho Falls, Idaho 83415

Phone: 208-526-0479

Facsimile: 208-526-3150

Additional file 1: Figure S1. Storage performance of *Scenedesmus acutus* biomass after 30 or 180 days of wet anaerobic storage. Error bars represent lower and upper bounds of approximate 95% confidence intervals. Points represent the observed mean in each category.


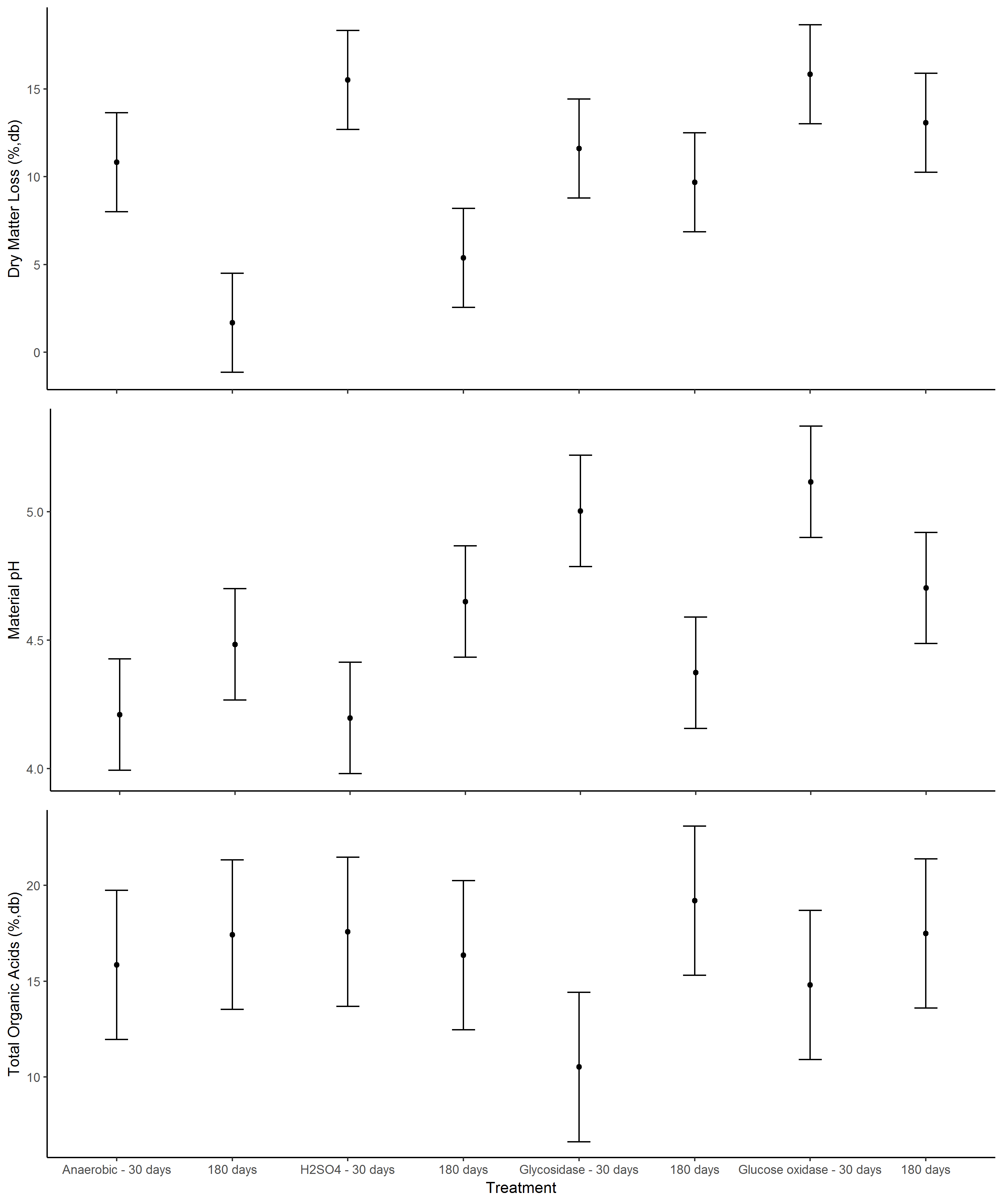


Additional file 1: Figure S2. Fatty acid composition of *Scenedesmus acutus* algae biomass (20% solids) after 30 or 180 days of wet anaerobic storage
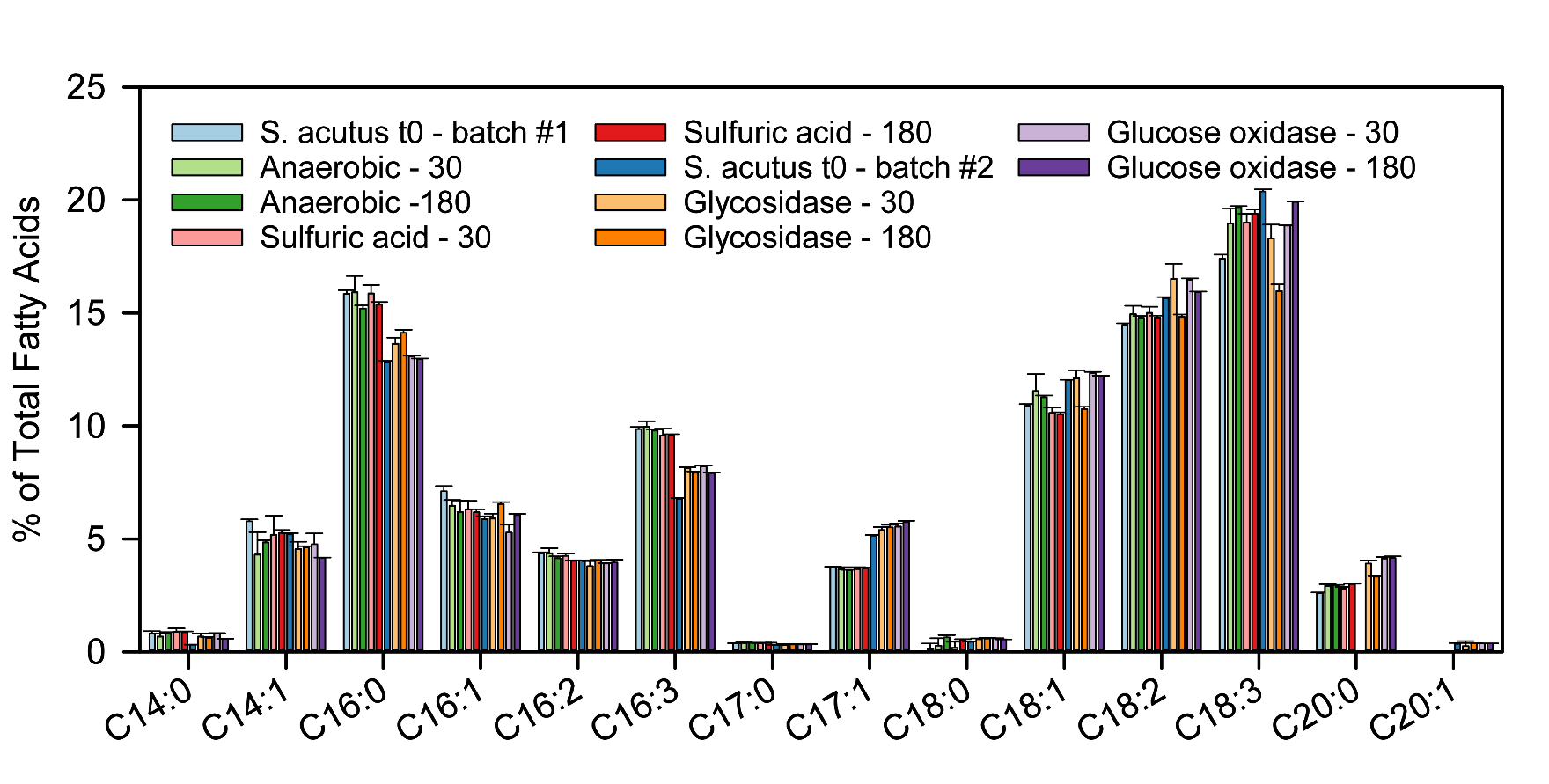


Additional file 1: Table S1. Proximate and ultimate analysis of *Scenedesmus acutus* algae biomass (20% solids) after 30 or 180 days of wet anaerobic storage

|  | | | | | | | | | | |
| --- | --- | --- | --- | --- | --- | --- | --- | --- | --- | --- |
| Experimental Condition | Length of Storage | Carbon  (%, db) | Hydrogen  (%, db) | Nitrogen  (%, db) | Oxygen  (%, db) | Sulfur  (%, db) | Volatiles  (%, db) | Ash  (%, db) | Fixed Carbon  (%, db) | HHV (BTU/g) |
| *S. acutus* – t_0_^1^ | 0 | 51.31 ± 0.07 | 7.22 ± 0.03 | 6.72 ± 0.02 | ND | ND | ND | ND | ND | 10003 ± 13 |
| Anaerobic | 30 | 51.05 ± 0.06 | 7.28 ± 0.03 | 6.57 ± 0.22 | ND | ND | ND | ND | ND | 10010 ± 6 |
|  | 180 | 53.05 ± 0.07 | 7.14 ± 0.03 | 6.72 ± 0.03 | 29.31 ± 0.11 | 0.27 ± 0.02 | 85.88 ± 0.20 | 3.50 ± 0.06 | 10.62 ± 0.19 | 10154 ± 24 |
| Sulfuric Acid 0.5% (db) | 30 | 52.57 ± 0.24 | 7.05 ± 0.06 | 7.04 ± 0.01 | 29.32 ± 0.30 | 0.26 ± 0.02 | 86.35 ± 0.30 | 3.75 ± 0.02 | 9.89 ± 0.32 | 10230 ± 54 |
|  | 180 | 53.22 ± 0.05 | 7.19 ± 0.02 | 7.19 ± 0.01 | 28.21 ± 0.05 | 0.30 ± 0.01 | 85.86 ± 0.06 | 3.89 ± 0.04 | 10.25 ± 0.09 | 10260 ± 4 |
| *S. acutus* – t_0_^2^ | 0 | 52.45 ± 0.09 | 6.90 ± 0.03 | 6.85 ± 0.02 | 30.01 ± 0.12 | 0.28 ± 0.01 | 84.62 ± 0.10 | 3.50 ± 0.08 | 11.87 ± 0.15 | 10099 ± 42 |
| Glycosidase | 30 | 54.89 ± 0.38 | 7.10 ± 0.05 | 7.51 ± 0.06 | 26.37 ± 0.48 | 0.31 ± 0.01 | 85.73 ± 0.14 | 3.81 ± 0.06 | 10.46 ± 0.19 | 10731 ± 56 |
|  | 180 | 56.40 ± 0.58 | 7.35 ± 0.10 | 8.26 ± 0.10 | 23.34 ± 0.78 | 0.29 ± 0.02 | 85.40 ± 0.14 | 4.36 ± 0.04 | 10.24 ± 0.15 | 10943 ± 37 |
| Glucose oxidase | 30 | 56.53 ± 0.05 | 7.19 ± 0.03 | 7.95 ± 0.01 | 24.05 ± 0.07 | 0.27 ± 0.01 | 85.49 ± 0.22 | 4.01 ± 0.07 | 10.50 ± 0.20 | 11093 ± 19 |
|  | 180 | 56.94 ± 0.08 | 7.32 ± 0.02 | 8.58 ± 0.02 | 22.65 ± 0.07 | 0.25 ± 0.01 | 85.68 ± 0.13 | 4.27 ± 0.02 | 10.05 ± 0.14 | 11242 ± 22 |
| HHV-Higher Heating Value  ^1, 2^ Harvest 1 and 2 | | | | | | | | | | |

Additional file 1: Table S2. Capital and operating costs utilized for dryers and dry and wet storage vessels.

| **Capital Costs, MM$** |  |
| --- | --- |
| Above-ground storage vessels (304SS) | $46.6 |
| In-ground storage pits (plastic lined) | $4.3 |
| Cover for in-ground storage pit (plastic cover) | $0.4 |
| Rotary drum dryer | $7.2 |
| Contact drum dryer | $20.4 |
| **Operating Costs, MM$/yr** |  |
| Rotary drum dryer | $1.6 |
| Contact drum dryer | $1.6 |

MM-Million; SS-Stainless steel

Additional file 1: Table S3. Discounted cash flow analysis parameters for the TEA.

| Plant life | 30 years |
| --- | --- |
| Discount rate | 10% |
| General plant depreciation | 200% declining balance |
| General plant recovery period | 7 years |
| Steam (turbine) plant depreciation | 200% declining balance |
| Steam (turbine) plant recovery period | 7 years |
| Federal tax rate | 35% |
| Financing | 40% equity |
| Loan term | 10-yr loan at 8% APR |
| Construction period | 3 years |
| First 12 months’ expenditures | 8% |
| Second 12 months’ expenditures | 60% |
| Third 12 months’ expenditures | 32% |
| Working capital | 5% of fixed capital investment |
| Start-up time | 6 months |
| Revenues during start-up | 50% |
| Variable costs incurred during start-up | 75% |
| Fixed costs incurred during start-up | 100% |
